# The Challenges of Regulating Artificial Intelligence in Healthcare: Comment on "Clinical Decision Support and New Regulatory Frameworks for Medical Devices: Are We Ready for It? - A Viewpoint Paper"

**DOI:** 10.34172/ijhpm.2022.7261

**Published:** 2022-09-19

**Authors:** Martin McKee, Olivier J. Wouters

**Affiliations:** ^1^Faculty of Public Health and Policy, London School of Hygiene & Tropical Medicine, London, UK.; ^2^Department of Health Policy, London School of Economics, London, UK.

**Keywords:** Regulation, Clinical Decision Support, Artificial Intelligence

## Abstract

Regulation of health technologies must be rigorous, instilling trust among both healthcare providers and patients. This is especially important for the control and supervision of the growing use of artificial intelligence in healthcare. In this commentary on the accompanying piece by Van Laere and colleagues, we set out the scope for applying artificial intelligence in the healthcare sector and outline five key challenges that regulators face in dealing with these modern-day technologies. Addressing these challenges will not be easy. While artificial intelligence applications in healthcare have already made rapid progress and benefitted patients, these applications clearly hold even more potential for future developments. Yet it is vital that the regulatory environment keep up with this fast-evolving space of healthcare in order to anticipate and, to the extent possible, prevent the risks that may arise.

## Regulating Health Products

 Regulation of health technologies must be rigorous, instilling trust among both healthcare providers and patients. Regulatory mechanisms have developed over time, with advances often following revelations of weaknesses in the regulatory process, such as those that allowed the teratogenic drug thalidomide to be prescribed in the 1960s.^1^ The principles underlying the regulation of pharmaceuticals have been extended to other medical technologies. Now, as described in the accompanying paper by Van Laere and colleagues about the use of clinical decision support systems, regulators are working out how to deal with applications using artificial intelligence in healthcare.^2^

 There are many challenges in regulating healthcare technologies. An obvious example is how to deal with the emergence of side effects not identified in the initial trials, either because they are rare, only develop after some time, or are only found in patients with characteristics not included in those trials. Other issues relate to biological agents where superficially minor differences in manufacturing processes can impact on safety and effectiveness^3^ and challenges in recruiting enough patients for trials of drugs treating rare conditions.^4^ There are, arguably, even greater challenges with medical devices. Regulators may differ in what they see as falling within their remit, which has resulted in weak control and supervision of medical devices in many settings. The performance of the device may also vary according to the skill and experience of the operator.

 Yet these hurdles are relatively minor compared to those involved in regulating the growing use of artificial intelligence in healthcare. As Van Laere and colleagues conclude in their viewpoint article, “designing a regulatory framework that achieves the right balance between promoting innovation and fast market access on one side and ensuring safety and quality on the other side is very challenging.” We agree, and in this commentary seek to complement their analysis by looking in more detail at some of the issues that arise in the regulation of artificial intelligence in healthcare.

## Artificial Intelligence in the Healthcare Sector

 First, it may be useful to set out the scope for applying artificial intelligence in the healthcare sector, as some readers may be unfamiliar with its key characteristics. In essence, it seeks to improve on the decision-making process undertaken by human operators as they synthesise and interpret information and make decisions. Recent advances have incorporated probabilistic reasoning to deal with uncertainty and machine learning, whereby the algorithms improve with experience. Machine learning now underpins most of modern artificial intelligence and can be unsupervised, seeking a pattern in the data presented to it, or supervised, whereby it learns from information fed into it by a human who has labelled it (for example, by adding the definitive diagnosis to a package of clinical data). Artificial intelligence can use a wide range of data inputs,^5^ although most of the early applications in healthcare relied on visual images, such as those used in radiology (eg, positron emission tomography scans), pathology (eg, images of cells and tissues), or ophthalmology (eg, retinal pictures).^6^ In due course this has expanded to the analysis of more complex three-dimensional images, such as those obtained at colonoscopy, and a wide range of physiological data, such as that generated by echocardiography, most often with the aim of making a diagnosis. In some cases, data are being linked in imaginative ways. For example, analysists have successfully combined data on symptoms with recordings of coughs to accurately diagnose respiratory infections,^7^ and linked radiographic and longitudinal clinical data to offer a prognosis and inform subsequent monitoring and treatment.^8^

 In these ways, artificial intelligence can not only improve the quality of care but, crucially, in health systems that are often constrained by numbers of skilled health professionals, also support increased activity. There is enormous potential to take advantage of the vast quantities of data that can now be collected on people engaged in everyday activities through wearable technology, such as the activity trackers contained within smartphones or devices that continuously monitor, for example, blood glucose levels.

 As with medicines and medical devices, it is not possible to make generalisations about the performance of clinical decision support software that rely on artificial intelligence, but there is now considerable evidence that, in certain circumstances, some can perform as well as, or even better, than human decisionmakers.^9-11^ Yet artificial intelligence is not a panacea, as recent experiences during the coronavirus disease 2019 (COVID-19) pandemic have shown.^12^ In 2007 Weiner and colleagues used the term “e-iatrogenesis” to denote patient harm resulting from information technology.^13^ Cabitza and colleagues have identified four broad risks.^14^ These are that artificial intelligence may deskill health workers, whose performance may be degraded if the product is unavailable or dysfunctional; it may fail to take account of context, such as differences in patient mix in different settings; it may fail to take account of uncertainty, for example in categorising input data that are subject to inter-observer variability; and problems may arise from the opacity of the process. Burrell^15^ identifies three aspects to this opacity. Two of these aspects, namely corporate secrecy by the provider and technical illiteracy by the user, can be overcome, at least in theory. But the third, the intrinsic complexity of the algorithm, cannot easily be addressed. Grote and Berens^16^ illustrate the problem with reference to the common situation where two expert clinicians disagree. They can discuss the reasons for their disagreement but where a clinician and a machine disagree the conversation will be one-sided.

 Finally, artificial intelligence is contributing to healthcare in other ways too. Biosimulation, in which the behaviour of chemical entities is analysed in silico, is becoming increasingly important in drug development.^17^ In transcriptomics, which is the study of messenger RNA to ascertain which of an organism’s genes are active, artificial intelligence is being used to analyse genomic and transcriptomic data from microorganisms to detect antimicrobial resistance.^18^

## Challenges Facing Regulators

 These developments have potentially profound consequences for clinical practice, but they also raise very difficult issues for regulators who are charged with protecting the public from unsafe and ineffective tools. We can identify at least five.

 First, an artificial intelligence application, where utilised, is only one part of a complex clinical system. It will require data to be inputted in an acceptable form. But what if the data input device is inadequately calibrated, or the application has been trained on high-quality images but is presented with low-quality ones? Will the regulatory process be able to take this into account?

 Second, the process of training the application may incorporate existing values and biases without making them explicit. For example, one study found that a white patient given a certain score by an application designed to estimate risk in primary care patients in the United States was deemed healthier than a black patient with the identical score.^19^ This was because the outcome variable was based in part on cost of treatment, with black patients typically receiving less expensive care. Biases in algorithms might be reduced by granting analysts access to larger, more representative datasets, but that would mean combining data from different providers into a single application. Regulation that enables this, while putting safeguards in place to ensure it is done safely, ethically, and in a manner that maintains individual privacy, could go a long way in improving artificial intelligence systems in healthcare.

 Third, where the application includes machine learning, its performance will change over time. This suggests that regulatory approval should be time limited. But how frequently should it be redone, given the trade-off between risk (which may be exceedingly difficult to estimate) and regulatory burden (which is measurable)? The US Food and Drug Administration has proposed a life-cycle process, from pre-market development to post-market performance, but how this will work out in practice is unclear.^20^

 Fourth, artificial intelligence in healthcare can conflict with data protection legislation, which in many settings (such as those covered by the European Data Protection Regulation) requires that only data required for the purpose intended should be collected. Yet artificial intelligence applications are extremely data hungry and it is often very difficult, if not impossible, to determine what information is necessary for the algorithms to function and what is not.^21^ It also raises issues of potential fraud: A recent World Health Organization (WHO) report has highlighted this danger, noting how a survey distributed by Facebook that was purported to be a psychological test was used to develop algorithms later used to influence elections.^22^ In the context of healthcare, should private health insurers be able to secure access to sensitive information that could help them predict the risk of individuals requiring healthcare, then they could use these data (which they are not supposed to have access to) to illegally adjust premiums. This could impact millions of individuals if done at scale. While this was always a potential concern with any illegal access to medical records, the opportunities created by artificial intelligence are immense.

 Fifth, applications are gathering vast quantities of data, raising issues of privacy. It is possible to identify characteristics of the patient that they do not want to be recorded in their data. It is known that artificial intelligence can predict parameters such as chronological age from even quite limited radiological data, something that is not especially surprising.^23^ However, Gichoya and colleagues have shown that deep learning algorithms can predict race with a high level of accuracy from a wide variety of radiological images.^24^

 These are some of the main issues facing regulators assessing artificial intelligence as a diagnostic aid. However, there is one other area that, although in its infancy, should not be overlooked. Earlier we mentioned its use in drug design. Yet, as was the case with other technologies, such as nuclear energy, things can be used for both good and evil. While algorithms used in this way are typically designed to screen out toxicity, a group of researchers turned this on its head. In a proof of concept study they showed that, within a few hours, they could design analogues of known chemical weapons predicted to be even more toxic.^25^ They call for greater awareness of the scope for dual use of artificial intelligence, ethics training for those involved, and channels for reporting potential abuses. In summary, this provides another, previously largely ignored challenge for those regulating artificial intelligence that falls on the margins of existing technology assessment models.

 Given these issues, it will often be difficult to decide who is accountable if things go wrong. When a medicine is approved, the approval comes with conditions. These include the indications for use (ie, the condition that the product is used for) and perhaps patient characteristics such as age or renal function which should be considered when administering a product, or other medications with which it should not be given because of known interactions. The physician can still use it if these conditions are not met, as an off-label prescription, but then takes responsibility. Of course, even with correct use, a medication administered may still be unsafe, perhaps because there was a risk that should have been identified during development but was not or it was not stored in the right conditions. In such cases, the responsibility is clear. The situation is much more difficult with artificial intelligence-based applications. Is it the designer of the initial algorithms, the person responsible for entering the data such as the echocardiograph operator, or the clinician proposing treatment who must decide how much weight to place on the answer given by the software when it conflicts with other evidence visible to the clinician but not captured by the algorithm that is responsible?

## What Can or Should Regulators Do?

 Van Laere and colleagues have described in detail the mechanisms that US and European authorities have put in place to regulate artificial-intelligence tools for clinical decision support.^2^ They outlined difficulties in defining which products should be subject to such regulation and classifying the risk profiles of products. In an accompanying commentary, Maresova expanded on the regulation around medical devices in the European Union.^26^ There is hope that the European Union’s proposed Artificial Intelligence Act, which would be the first comprehensive regulatory scheme for artificial intelligence worldwide and would divide products into risk categories, will usher in a new era in the regulation of artificial intelligence and establish a global standard for regulators and manufacturers.^27^

 We agree with Van Laere and colleagues that neither set of regulators have yet clarified all of the issues that arise. Among the concerns that they raise, we see two as being especially challenging. The first, which has also been raised by the US Food and Drug Administration, is the need to devise a regulatory process that spans the entire life-cycle of the application.^28^ This should be one that fosters innovation while ensuring patient safety, a difficult balance to achieve. It will require development of standards at all points on this cycle. Starting with the premarket authorisation, this would include the creation of a process similar to the phases of clinical trials undertaken with pharmaceuticals, although adapted to the different context. For example, this might include standards for validation of the algorithms, although given the many ways in which artificial intelligence can be used, we should not underestimate the challenges of doing this. While, superficially, it would seem that the ability to explain the logic would be desirable, as the WHO report noted, this risks inhibiting innovation.^22^ However, there is an argument for including a requirement that algorithms should be capable of being evaluated independently. It would also include a test of patient benefit that would screen out applications that are really only data harvesting tools. Once the application has been introduced, there should be clear rules for when changes to the software were of sufficient importance to justify a further review to ensure that new risks had not been introduced. Finally, there could be a requirement for regular audits to be undertaken, at prespecified intervals, to identify potentially hazardous drift from the initial performance.

 The second is one that is common to the regulation of conventional medical products, including pharmaceuticals. This is how the evaluations that inform the regulatory process may be conducted in populations that are unrepresentative of those on whom they will ultimately be used. For example, it is well known that many clinical trials of medicines exclude older people or those with multi-morbidity.^29^ This is even more important when evaluating artificial intelligence applications. Furthermore, it is especially important to anticipate, investigate, and prevent algorithms from replicating or reinforcing existing biases. This depends on a high level of awareness of the risks but can be mitigated by measures such as the creation of training datasets that have been evaluated as having low risk of bias.^30^

 None of this will be easy. While artificial intelligence applications in healthcare have already made rapid progress, these applications clearly hold even more potential for future developments. The innovations that they have already provided are bringing benefits to patients. However, the regulatory environment needs to keep up with this fast-evolving space of healthcare in order to anticipate and, to the extent possible, prevent the risks that may arise.

## Ethical issues

 Not applicable.

## Competing interests

 Authors declare that they have no competing interests.

## Authors’ contributions

 Both authors contributed equally to this work.
